# Maternal iodine status during lactation and infant weight and length in Henan Province, China

**DOI:** 10.1186/s12884-017-1569-0

**Published:** 2017-11-16

**Authors:** Jin Yang, Lin Zhu, Xiaofeng Li, Heming Zheng, Zhe Wang, Zongyu Hao, Yang Liu

**Affiliations:** Department for Endemic Disease Control and Prevention, Center for Disease Control of Henan Province, Room 4201, No. 105 Nongye Nan Road, Zhengdong New District, Zhengzhou, 450016 China

**Keywords:** Iodine, Lactation, Height for age, Weight for age, Maternal nutrition

## Abstract

**Background:**

Infants are very sensitive to iodine deficiency. Breastfed infants are dependent on maternal iodine intake. The aim of this study was to evaluate the relationship between maternal iodine status during lactation and infant weight and length.

**Methods:**

A cross-sectional survey was conducted to investigate maternal iodine status and infant anthropometric measures in Henan Province, China. Only exclusive breastfeeding mothers and their infants < 6 months of age (*n* = 747) were included in our final analysis. Urine samples were collected from all the mothers and infants. Infant weight and length were measured and converted into weight-for-age Z-score (WAZ) and height-for-age Z-score (HAZ) using the World Health Organization (WHO) AnthroPlus software.

**Results:**

The median urinary iodine concentration (UIC) in lactating women was significant lower than that in their infants (177.4 vs 261.1 μg/L, *P* < 0.001). A positive correlation was found between maternal and infant urinary iodine concentration (*r* = 0.203, *P* < 0.01). The mean HAZ and WAZ values were lowest in the infants whose mothers had UIC below 50 μg/L (*n* = 41). Infant WAZ with maternal UIC below 50 μg/L was significantly lower than those with maternal UIC of 50 μg/L or above (*P* = 0.043). After adjusting for confounding factors, there were significant differences in infant WAZ between maternal UIC groups.

**Conclusions:**

The present study suggests that maternal iodine status during lactation may be related to their infant anthropometric index. Appropriate iodine intake of lactating women is beneficial for their infants.

## Background

Infants are very sensitive to iodine deficiency because of their high demands and low storages of iodine [1, 2]. The first 2 years of age is a crucial time for neurological development and growth. Even mild iodine deficiency may lead to irreversible damage during this period, such as low intelligence, neonatal hypothyroidism, short stature, skeleton disorders, and other growth retardation [2, 3]. It is well known that iodine is an essential material in synthesis of thyroid hormones (TH) [1, 3]. TH can regulate protein production, fat metabolism, and glucose utilization [4, 5]. TH also influences bone development by TH receptors in bone cells and the hypothalamic-pituitary-thyroid axis [6, 7]. Thereby, TH is closely involved in infant growth.

Infant iodine status is influenced by many factors, among which feeding type is the most important one. The difference in iodine status between breastfed and formula-fed infants was reported in numerous studies, but these results were conflicting. Remer T et al. observed that breastfed infants were more inclined to suffer from iodine insufficiency [2]. Gordon J et al. demonstrated that there was no difference in iodine status between breastfed and formula-fed infants in Boston [8]. Our previous study found that the median urinary iodine concentration (UIC) in breastfed infants was significantly higher than that in formula-fed infants, which was probably due to universal salt iodization in China [9].

Breastfed infants depend on maternal iodine intake. During lactation, mothers transfer iodine to their infants by mammary glands, which concentrate iodine in breast milk. Infants approximately require 7 μg I/kg of body weight to build iodine pool in their thyroid gland [10]. In order to ensure that infants get enough iodine, lactating mothers are recommended to consume iodine 250 μg/day [11].

Iodine deficiency has long been known as a risk factor for retard growth during childhood [12]. Evidence suggested that maternal iodine deficiency and hypothyroidism during pregnancy adversely affected neurodevelopment and intellectual capacity of their offspring [13–15]. Many studies also assessed the effects of maternal iodine deficiency and hypothyroidism on pregnancy outcomes, such as intrauterine growth restriction, fetal death, preterm, and low birth weight [16–18]. Previous studies of infant-mother pairs were limited by small sample size, not adjusting potential confounding factors. For example, an Argentina study was restricted by a small sample size (*n* = 77) [19]. Furthermore, few studies focused on the iodine status of lactating mother and infant size. The objective of the present study was to assess the relationship between maternal iodine status and infant anthropometric index.

## Methods

### Subjects

This study was conducted in Henan Province of China, where more than 90% of the households accessed to iodized salt. There are 18 cities in Henan Province. Subjects were recruited using a multi-stage stratified random sampling from April 2014 to September 2014. First, two counties were selected from each city in Henan Province. Second, a maternity clinic was selected within each chosen county. Finally, about 50 infant-mother pairs were sequentially enrolled in each chosen clinic. Inclusion criteria were: 1) full term and singleton birth; 2) no disease related to retarded growth in infant; 3) breastfed infant; 4) without the use of iodine-containing disinfectants; 5) no history of thyroid disease in mothers. A total of 1598 infant-mother pairs were sequentially enrolled. For the reason that complementary foods in the infants of > 6 months age might influence infant iodine intake and their size, only exclusive breastfeeding mothers and their infants < 6 months of age (*n* = 747) were included in final analysis.

The mothers were asked to complete a questionnaire, including current infant feeding practices, maternal age, education, socioeconomic status, health and obstetric history, iodine supplement, and parental height and weight.

The medical ethics committee at Henan Provincial Centre for Disease Control and Prevention approved the study. Written informed consents were obtained from all the mothers.

### Laboratory measurement

Urine samples were collected from both mothers and their infants. The samples were sealed in plastic bottles and refrigerated at 4 °C until analysis. UIC was measured to determine iodine in urine by the acid digestion method (As3 + −Ce4+ catalytic spectrophotometry), a national standard method developed by China’s Ministry of Health (WS/T107–2006) [20]. All urine samples were tested by the municipal CDC laboratories. External quality control was provided by China National Iodine Deficiency Disorders Reference Laboratory. Based on the results, the coefficient of variation for UIC in our laboratory was 2.0% at 68.2 ± 1.3 μg/L and 0.9% at 193.0 ± 10.0 μg/L. According to WHO standard for lactating women and infants, they are defined as iodine deficiency when the median UIC falls below 100 μg/L; 100 μg/L or higher as sufficient [21].

### Anthropometric measurement

Infant weight and length were measured according to a standard procedure. Infant wearing light clothes only were weighed using an electronic scale with 50 g precision. Infants in bare feet were laid down on a horizontal length measuring instrument with 0.1 cm precision. The trained staff moved the sliding foot piece to infant feet and read the recumbent length. Height-for-age Z-score (HAZ) and weight-for-age Z-score (WAZ) were calculated for each infant by using WHO-Anthro software (http://www.who.int/childgrowth/software/en/).

### Statistical analysis

Normally distributed data was summarized as the mean ± standard deviation (SD). Non-normally distributed data was presented as the median with interquartile range (IQR). Independent t-test or ANOVA was used to compare the mean anthropometric index between different groups. Wilcoxon or Mann–Whitney U test was used to test the difference in UIC between different groups. Chi- square test was used to compare the difference of categorical variables. Pearson correlation was performed for the correlation between maternal and infant UIC. A multiple linear regression analysis was conducted with infant HAZ or WAZ as dependent variable and maternal UIC as the main independent variable, adjusting for other covariates, including maternal socioeconomic factors, age, education, and parental height and weight. In order to reflect the direct effect of maternal UIC on infant anthropometric indexes, only women whose infant had UIC within the normal range (50-299 μg/L) were included in multiple regression analyze (*n* = 451). All analyses were conducted using SPSS version 16.0. Statistical significance was defined as *P* < 0.05.

## Results

### Characteristics of mothers and infants

Maternal and their infant characteristics (*n* = 747) were shown in Table 1. 67.0% of the mothers reported incomes of less than 3000 yuan per month, and only 27.5% of them had high school education. About 29.3% of the mothers used iodine supplements during lactation. The mean age of the infants was 3.6 ± 1.8 month, and 57.8% of them were infant boys.

**Table 1:** Characteristics of mothers and their infants (*n* = 747)

Maternal and infant variables	values
Mothers	
Age (Mean ± SD, yr)	27.4 ± 4.3
Education	
Primary or less than primary (%)	48.8
Secondary (%)	23.6
High school or above (%)	27.5
Month income	
< 1000 yuan (%)	9.4
1000–2999 yuan (%)	57.6
≥ 3000 yuan (%)	33.0
Maternal occupation	
Unemployed/housewives	43.1
Agriculture	22.9
Manual	8.9
Sales and services	3.2
Professional/technical/managerial	12.6
others	9.3
Height(Mean ± SD)	161.0 ± 4.2
Weight(Mean ± SD)	58.6 ± 8.8
BMI(Mean ± SD)	22.6 ± 3.2
UIC (Median (IQR), μg/L)	177.4 (116.1–267.8)
UIC < 50 μg/L (%)	5.4
UIC < 100 μg/L (%)	19.0
Infants	
Age (Mean ± SD, month)	3.6 ± 1.8
Male (%)	57.8
UIC (Median (IQR), μg/L)	261.1 (172.7–366.1)
UIC < 50 μg/L (%)	2.7
UIC < 100 μg/L (%)	7.8
HAZ(Mean ± SD)	−0.008 ± 2.2
WAZ(Mean ± SD)	0.4 ± 1.6

*SD* standard deviation, *IQR* interquartile range, *UIC* urinary iodine concentration, *HAZ* height-for-age Z-score, *WAZ* weight-for-age Z-score, *BMI* body mass index

### UIC of mothers and their infants

The median UICs in mothers and their infants (*n* = 747) were 177.4 μg/L (IQR 116.1–267.8) and 261.1 μg/L (IQR 172.7–366.1), respectively, which both met WHO criterion of iodine sufficiency (>100 μg/L). Maternal UIC was significantly lower than that in their infant (*P* < 0.001). In addition, the percentages of mothers and their infants with UIC above 100 μg/L were 81.0% and 92.7%, respectively. The mothers consuming iodine supplements had higher UIC than those who did not consuming iodine supplements (191.7 vs 173.8 μg/L, *P* < 0.01). The median UIC in infant boys was significantly higher than that in infant girls (267.7 vs 244.8 μg/L, *P* < 0.01).

### Relationship between maternal UIC and infant UIC

30.4% of the mother-infant pairs (*n* = 227) had the same categories of UIC (Table 2). A positive correlation was found between maternal and infant UIC as continuous variables (*r* = 0.203, *P* < 0.001).

**Table 2:** Distributions of maternal and infant UIC

Distribution of Maternal UIC	Distribution of Infant UIC (n (%))				
	< 50 μg/L	50–99 μg/L	100–199 μg/L	200–299 μg/L	> 299 μg/L
< 50 μg/L	3(7.5)	6(15.0)	9(22.5)	10(25.0)	12(30.0)
50–99 μg/L	3(3.0)	10(9.9)	27(26.7)	33(32.7)	28(27.7)
100–199 μg/L	6(2.1)	16(5.6)	89(30.9)	80(27.8)	97(33.7)
200–299 μg/L	3(1.7)	2(1.2)	42(24.4)	55(32.0)	70(40.7)
≥ 300 μg/L	1(0.7)	4(2.7)	31(21.2)	40(27.4)	70(47.9)

Maternal UIC was positively correlated with infant UIC (*r* = 0.203, *P* < 0.001)

*UIC* urinary iodine concentration

### Relationship between maternal UIC and infant anthropometric measures

In maternal UIC (Table 3), the mean HAZ and WAZ values were lowest in the infants whose mothers had UIC below 50 μg/L (*n* = 41). Analyzed by ANOVA, HAZ and WAZ had no significant difference between different maternal UIC groups (*P* > 0.05).

**Table 3:** Anthropometric indices of the infants according to the distributions of maternal UIC

Maternal UIC	Infant HAZ (Mean ± SD)	Infant WAZ (Mean ± SD)
< 50 μg/L	−0.36 ± 1.78	0.12 ± 1.47
50–99 μg/L	0.22 ± 2.40	0.57 ± 1.64
100–199 μg/L	0.12 ± 2.21	0.40 ± 1.61
200–299 μg/L	−0.23 ± 2.24	0.41 ± 1.51
> 299 μg/L	−0.05 ± 2.10	0.33 ± 1.77
*P* value^a^	0.296	0.614

*SD* standard deviation, *UIC* urinary iodine concentration, *HAZ* height-for-age Z-score, *WAZ* weight-for-age Z-score, *BMI* body mass index

^a^ANOVA was used to compare the mean anthropometric index between different groups

When all the categories with maternal UIC of 50 μg/L or above were combined, the mean HAZ, and WAZ values were −0.02 ± 2.22, and 0.41 ± 1.61, respectively. Analyzed by *t* test, the infant WAZ value with maternal UIC below 50 μg/L (*n* = 41) was significantly lower than those with maternal UIC of 50 μg/L or above (*P* = 0.043), whereas infant HAZ did not show statistical significance between two maternal UIC categories.

### Multiple regression analysis

In the multiple regression model with infant HAZ as dependent variable (table 4), infants with maternal UIC below 50 μg/L had slightly lower HAZ values than infants with maternal UIC between 50 and 99 μg/L.

**Table 4:** Adjusted associations between maternal UIC and infant HAZ, WAZ, and BMI, using multiple linear regression^c^

Maternal UIC	HAZ^a^		WAZ^b^	
	Adjusted coefficient	*P* value	Adjusted coefficient	*P* value
< 50 μg/L	Reference		Reference	
50–99 μg/L	0.965	0.076	0.918	0.011
100–199 μg/L	0.794	0.121	0.671	0.038
200–299 μg/L	0.524	0.424	0.779	0.031
> 299 μg/L	0.795	0.208	0.800	0.027

^a^Independent variables entered in the regression were maternal UIC, maternal age, education, occupation, and income, and maternal and parental height. Dependent variable was infant HAZ

^b^Independent variables entered in the regression were maternal UIC, maternal age, education, occupation, and income, and maternal BMI. Dependent variable was infant WAZ

^c^Only women whose children had UIC within the normal range (50-299 μg/L) were included (*n* = 450)

In the multiple regression model with infant WAZ as dependent variable, maternal occupation, and paternal weight showed a significant association with WAZ. After adjusting for maternal BMI and other maternal socioeconomic factors, infants with maternal UIC below 50 μg/L had significantly lower WAZ than those with maternal UIC above 50 μg/L.

## Discussion

In our study, the median UICs of mothers and their infants were 176.8 μg/L and 261.1 μg/L, respectively, and they were regarded as “iodine sufficient” according to WHO criterion [21]. The results can be attributed to the universal salt iodization in China in 1995. Based on the national survey in 2014, the household coverage rates with iodized salt was 93.3% in Henan Province. Some studies found that the median UIC in lactating women might be less than that in pregnant women, which was partly due to the iodine loss into breast milk and the normalization of thyroid hormone production [22–26]. Our research indicated that infant UIC was higher than maternal UIC. The possible reason is that thyroid and renal functions in infants are not immature and therefore excess iodine can not be retained.

Breastfed infants mainly obtain iodine from breast milk. The mammary glands have better ability in concentrating of iodine by the activities of sodium/iodine symporter and deiodinase [27]. In order to meet the needs of infant growth and development, the concentration of breast milk should be maintained between 100 and 200 μg/L [28, 29]. Evidence showed that breast-milk iodine concentration (BMIC) was correlated with UIC [30, 31]. Due to the limitation of experimental condition and easily accessibility, maternal UIC were used as a proxy of BMIC in our study. A few studies indicated that infant UIC was affected by their mothers [32, 33]. Our finding showed that maternal UIC had a positive but weak correlation with infant UIC (*r* = 0.203*, P* < 0.001).

WHO-recommend daily intakes for lactating women and infants are 250 and 90 μg I/d, respectively [11]. Although iodine supplements could supply adequate iodine intake during lactation and infancy, WHO and European pediatric societies do not recommend iodine supplements for infants or their mothers in iodine-sufficient regions [34, 35]. In contrast, some reports from the United States [36], the Switzerland [24], and the New Zealand [37], recommend that lactating women should receive 150 μg I/d and iodine fortification of infant food should be encouraged. In our research, iodine-supplemented mothers had higher UIC than unsupplemented mothers (191.7 vs 173.8 μg/L, *P* < 0.01). Morse [38] proved that maternal supplementation of vitamin D, DHA, folic acid and iodine during pregnancy and lactation can be beneficial for brain development of their offspring.

Adequate iodine intake is important for the growth and development of fetus and infant [39]. A review reported that there was a connection between the use of iodized salt and the increased weight and arm circumference of infant, especially in the second year of life [40]. Alvarez-pedrerol et al. [41] stated that the UIC of pregnant women in the third trimester was positively correlated with birth weight of their offspring. An Argentina research [19] demonstrated that mothers with lower UIC had lower placental weight and head perimeter in their newborns. For exclusively breastfed infant, breast milk is the main source of iodine. Sufficient iodine status of lactating women is essential for their infant growth and maturation. In the present study, the lowest mean values of HAZ and WAZ were found in infants whose mothers had UIC below 50 μg/L. After adjusting for maternal age, parental weight and height, and other socioeconomic factors, we observed that maternal UIC had positive effect on infant weight. A similar model demonstrated that the mean weight and length of boys increased by 70 g and 0.41 cm for every 0.5 mg/L increase in maternal UIC [42]. Fisher [43] reported that a moderate iodine deficiency in lactating women resulted in a 30% reduction of thyroxine in their infants, which played an important role in bone development. For another view, a study from Korea [44] found that excessive iodine intake of lactating women may be linked with hypothyroidism in their preterm infants. Infant with hypothyroidism may experience intellectual and growth retardation. These facts may be explained why maternal iodine affects infant anthropometric indices.

In our study, UIC was divided into five groups instead of a continuous variable, which could decrease the misclassified possibility due to high variation of UIC [39]. Although UIC only represents recent iodine intake in an individual, low iodine status could exist a long time in an individual [45]. In addition, we used HAZ and WAZ to replace the original values of height and weight in order to avoid the differences of infant gender and age. The major limitation was that UIC was not adjusted for the creatinine clearance. The ratio of urinary iodine and creatinine can reflect the real iodine intake of individuals by excluding the influence of urinary capacity.

## Conclusions

In conclusion, our research indicates that the iodine status of breastfeeding mothers may be related to their infant weigh. Appropriate iodine intake of lactating women is beneficial for their infants.

## Abbreviations

BMI: Body mass index
BMIC: Breast-milk iodine concentration
HAZ: Height-for-age Z-score
IQR: Interquartile range
SD: Standard deviation
TH: Thyroid hormones
UIC: Urinary iodine concentration
WAZ: Weight-for-age Z-score
WHO: World Health Organization
